# Rotational Guided Growth: A Preliminary Study of Its Use in Children

**DOI:** 10.3390/children10010070

**Published:** 2022-12-29

**Authors:** Dror Paley, Claire Shannon

**Affiliations:** Paley Orthopedic and Spine Institute, West Palm Beach, FL 33407, USA

**Keywords:** guided growth, femoral anteversion, tibial torsion, rotational malalignment, miserable malalignment, growth modulation, hemiepiphysiodesis, growth tether

## Abstract

Torsional malalignment of the legs is common in children, and those that do not remodel may benefit from surgical correction. Traditionally, this is corrected with an open osteotomy. Guided growth is the gold standard for minimally invasive angular correction and has been investigated for use in torsional deformities. This study presents our preliminary results of rotationally guided growth in the femur and tibia using a novel technique of peripheral flexible tethers. A total of 8 bones in 5 patients were treated with flexible tethers consisting of separated halves of a hinge plate (Orthopediatrics Pega Medical, Montreal, QC, Canada), which were fixed to the epiphysis and metaphysis at 45° angles to the physis and connected with Fibertape (Arthrex, Naples, FL, USA). The implants are placed medially and laterally in the opposite 45° inclination, determined by the desired direction of rotation. Additionally, the average treatment time was 12 months. All patients corrected the rotational malalignment by clinical evaluation. The average rotational change was 30° in the femurs and 9.5° in the tibias. Further, the average follow-up was 18 months, with no recurrence of the rotational deformity. There was no change in longitudinal growth in the patients who underwent bilateral treatment. Rotational guided growth with flexible tether devices is a novel technique that successfully corrects torsional malalignment without invasive osteotomy surgery.

## 1. Introduction

Torsional malalignment of the lower extremities is common in children. Most patients present because their parents complain that their child is in-toeing or out-toeing while walking. They may also complain that they fall more often because of this. At older ages, they may complain of hip, knee, or ankle pain associated with in- or out-toeing. Those who do not remodel with growth may benefit from surgical correction. Traditionally, the only surgical technique available for treating rotational deformities of the femur or tibia was osteotomy, requiring open surgery, a period of non-weightbearing, and frequently, an inpatient hospital stay. The use of guided growth has been of great interest in the treatment of torsional deformities due to the decreased morbidity and limited recovery time that have been demonstrated in the treatment of frontal plane deformities [1]. A minimally invasive technique was recently reported as a method to correct torsional deformities in the femur in children [2] using a circumferential cable. The purpose of this study is to report the preliminary results of a novel technique to treat rotationally guided growth in the femur and tibia using counter-opposed, crossed, inclined peripheral flexible tethers.

## 2. Materials and Methods

**Figure 1:**
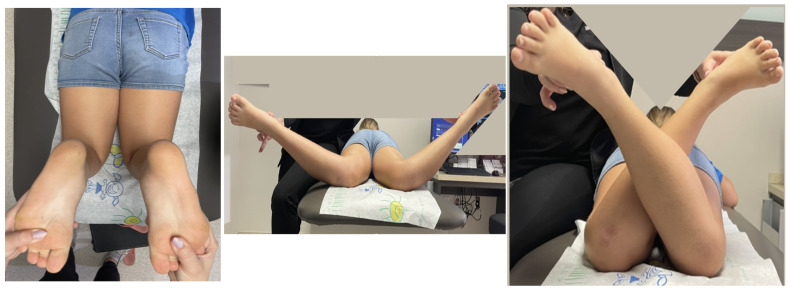
Prone clinical evaluation of the thigh-foot axis (**left**). Prone measurement of internal femoral rotation (**middle**) and external femoral rotation (**right**) (Patient #1 preoperatively).

The institutional review board’s approval was obtained. Torsional deformity was defined as a positive (external) or negative (internal) foot progression angle outside the normal physiologic parameters (+5 to +15 degrees) [3,4]. Once a torsional deformity was identified and determined to be symptomatic by history, a physical examination was performed to quantify the rotational profile. The tibial rotation was measured using a goniometer to measure the prone thigh-foot axis. Additionally, femoral rotation was measured using a goniometer in the prone hip internal and external rotation profile according to Staheli [3] (Figure 1).

A total of five patients with torsional deformities in eight bone segments (femur 5, tibia 3) were treated with flexible counter-opposed crossing tethers using the surgical method described. Two of the patients underwent bilateral distal femoral rotationally guided growth for idiopathic bilateral femoral anteversion. One patient underwent two ipsilateral rotational guided growth surgeries, first on the femur and then on the tibia, for internal torsional deformity. Additionally, the other two patients underwent unilateral rotationally guided growth to correct tibial rotation, one with internal torsion and one with external torsion. The demographics, preoperative, and postoperative rotational profiles for all included patients are listed in Table 1.

### Surgical Technique

**Figure 2:**
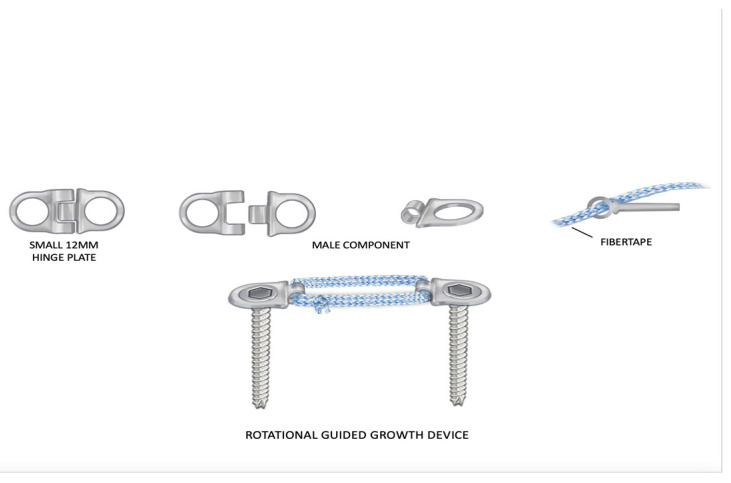
Illustration of the components of the rotationally guided growth implant used. Two of the same halves of the hinge plate were used. They were tethered together with fibertape. They were fixed to the bone with screws. Illustrations copyrighted to the Paley Foundation; reprinted with permission.

**Figure 3:**
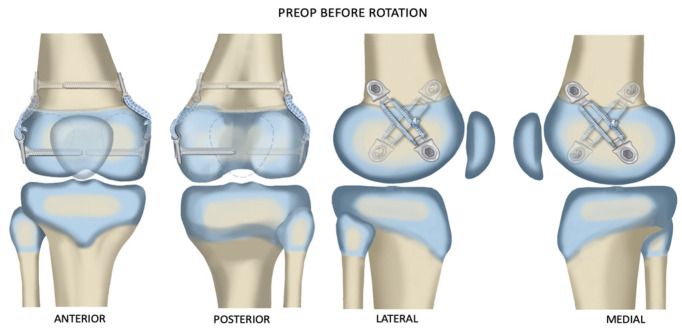
One implant was placed medially and the other laterally inclined, as shown, so that the crossing angle was as close to 90° as possible. Illustrations copyrighted to the Paley Foundation; reprinted with permission.

The Hinge Plate (Orthopediatrics, Pega Medical, Montreal, QC, Canada) is a hemi-epiphysiodesis screw-plate device that consists of two plate halves connected by a hinge. The hinge rivet is removed, and the two plate halves are separated (Figure 2). Only the male half of the plate is used. The separated halves are fixed into the epiphysis and metaphysis based on the desired rotational orientation and secured with a proprietary screw. In addition, the sections of plate are connected with Fibertape (Arthrex, Naples, FL, USA) to create a flexible tether. The fibertape is looped twice between the ends of the hinge plate halves, going through the hole where the hinge rivet was located. The fibertape ends are tied taut with five knots. Further, the lines created by the fibertape on each side of the bone should be at 45° to the long axis of the bone and perpendicular (90°) to each other (Figure 3. The two incisions are then closed. The patients are allowed to resume full weight-bearing and return to all activities as tolerated without restrictions.

Marks are made on the skin in line with the planned placement of the fixation on the medial and lateral sides of the distal femur or proximal tibia using the image intensifier. The two lines are made at opposite 45° angles to the long axis of the bone. In addition, when superimposed, the lines should cross each other at an angle of 90°.

**Figure 4:**
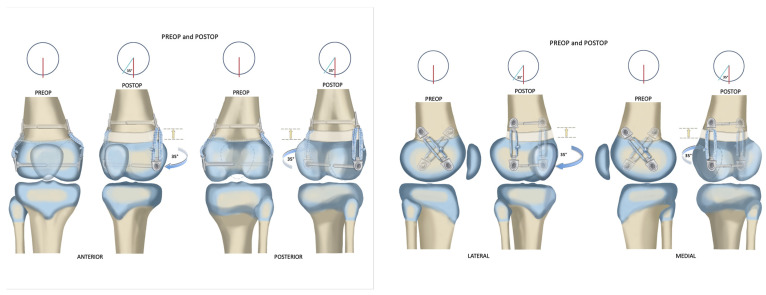
Anterior, posterior, lateral, and medial views of correction before and after rotationally guided growth correction. lllustrations copyright to Paley Foundation; reprinted with permission.

The orientation of the implant angle on each side of the bone depends on the direction of rotational correction desired. Therefore, on the right distal femur, to correct excessive internal rotation of the knee relative to the hip, the medial epiphyseal screw is placed posterior to the metaphyseal screw. The lateral epiphyseal screw is placed anterior to the metaphyseal screw (Figure 4). In addition, the medial epiphyseal screw is placed anterior to the metaphyseal screw, and the lateral epiphyseal screw is placed posterior to the metaphyseal screw for the right proximal tibia, to externally rotate the foot relative to the knee. Furthermore, to correct excessive external rotation of both the right distal femur and proximal tibia, the above screw orientations would be reversed. The medial epiphyseal screw is placed posterior to the metaphyseal screw, and the lateral epiphyseal screw is placed anterior to the metaphyseal screw in order to correct internal rotation of the left distal femur. Additionally, in the left internal tibial torsion, the medial epiphyseal screw is anterior and the lateral epiphyseal screw is posterior to their metaphyseal screws. There is no fibular fixation used for rotational correction of the tibia in either direction. The previous pattern is reversed for the left external tibial torsion.

## 3. Results

The average age at the time of rotational plate insertion was 8 years and 5 months (range: 2 years and 6 months–15 years and 7 months). The total number of patients had open growth plates on preoperative radiographs. The underlying diagnoses included idiopathic femoral anteversion (3), internal tibial torsion (1), healed congenital pseudarthrosis of the tibia with NF-1 (1), and congenital femoral deficiency (1).

**Figure 5:**
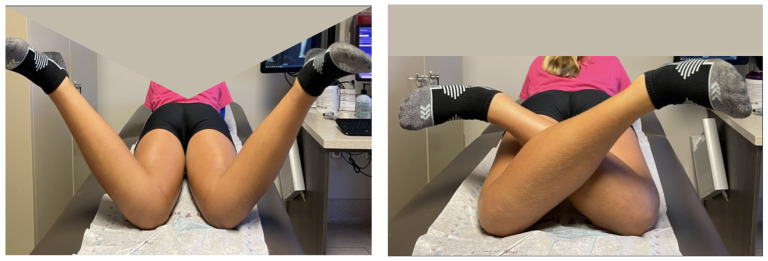
Postoperative internal (**left**) and external (**right**) rotation of both hips after rotationally guided growth correction. Compare this to the preoperative photos of the same patient in Figure 1 (Patient #1).

The post-operative rotational change was observed in all 8 bones treated. The average change in the femoral rotation patients was 30° (range 10°–45°). Additionally, the average change in the tibial rotation patients was 9.5° (ranging 5° to 17°). Further, the average time to correction was 11.8 months (range 7–18 months). Two of the patients underwent staged removal of the devices, removing the lateral plate tethers and leaving the medial plate tethers in place to correct residual genu valgum deformity. These plates have been subsequently removed. The average follow-up after plate removal was 18 months (range 2–33 months). Loss of rotational correction was not observed during this follow-up time. Moreover, the longitudinal growth during the time of rotational correction was evident but could not be compared to a contralateral normal side in the 3 patients (4 rotation guided growths) who underwent unilateral correction due to pre-existing leg length discrepancy (LLD). Two patients who did not have a LLD underwent bilateral treatment, and longitudinal growth remained the same on both sides. We were unable to determine if any slowing of longitudinal growth occurred as a result of the bilateral physeal tethering. There was no evidence of alteration of the posterior proximal tibial angle (PPTA) or posterior distal femoral angle (PDFA). The total number of patients returned to their preoperative level of activity after plate insertion and final plate removal (Figure 5).

## 4. Discussion

Hemiepiphysiodesis has evolved from the use of staples with perpendicular fixed legs to the use of a plate with pivoting screws [1]. The principle in both is to create a peripheral tether outside the physis, causing the physis to grow at its normal rate at the point farthest from the tether while limiting or temporarily stopping the growth at the point closest to the implant. This process has been renamed “guided growth” [5]. Until recently, the application of guided growth was to create an angular change in the frontal, sagittal, or oblique planes. Guided growth plates have also been used to create growth stoppage by epiphysiodesis [6]. This requires the placement of implants on opposite sides of the physis. It has been posited that the placement of plates on opposite sides of the growth plate at an inclined angle would lead to rotational tethering of growth before epiphysiodesis. This was corroborated in small animals (rabbits) by Arami et al., Sevil-Kilimici et al., and Lazarus et al. [7,8,9]. It was also corroborated in large animals (calves) by Martel et al. [10]. Most recently, it was also demonstrated to work in humans by Metaizeau et al. [2]. On the basis of these reports as well as this preliminary study, it is evident that rotationally guided growth can be achieved by two counter-opposed, obliquely oriented tethers on either side of the physis. The previously reported method used in animals and humans is a cable going through the bone and around the sides [2,7,10]. An alternative method was reported in rabbit studies using two inclined plates on opposite sides of the bone with no fixed structure passing through the bone from one side to the other [9]. The concern with the first method is that it can cause direct damage to the physis as the pressure of the cable cuts into the side of the bone between the epiphysis and metaphysis. The only human study reported knee stiffness as a common complication [2]. The disadvantage of the second method using inclined plates is that the plates are very stiff and nonmalleable, which could be prominent or restrictive as rotation occurs [9]. In this study, we used a tether without passing from one side of the bone to the other. The anchorage points to the bone were tethered using a flexible, soft material (fibertape), which lay on the surface of the physis. This may be safer and less abrasive to the physis than a taut metal cable.

**Figure 6:**
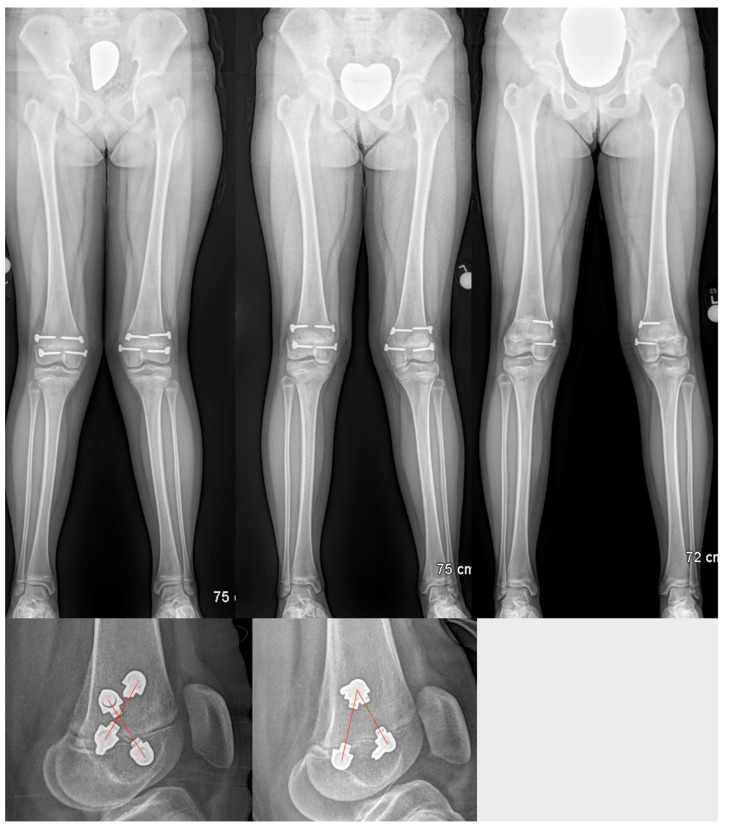
Standing radiographs of both lower limbs of the same patient as in Figure 1 and Figure 5 (Patient #1) at the time of insertion of rotational guided growth implants (**top left**), end of rotational guided growth (**top middle**), and medial hemiepiphysiodesis with the medial implant to correct valgus (**top right**). The crossing angle of the plates is seen at the beginning (**lower left**) vs. at the end (**lower right**) of the correction. Note the change in crossing angle.

**Figure 7:**
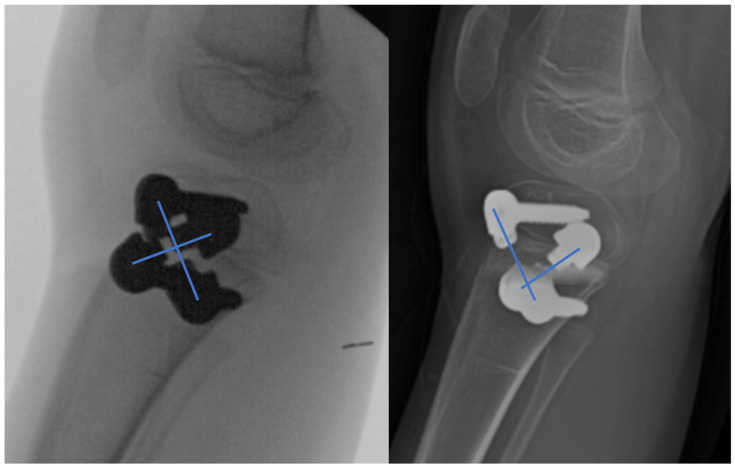
Rotationally guided growth of the tibia (patient #3). Lateral radiographs at the beginning (**left**) and end (**right**) of correction. Note the change in crossing angle. The tibial correction in this case was 17°.

Growth retardation was reported in rabbits by Lazarus et al. (mean 4.2%) [9] and Arami et al. [7] (mean 7%) in rabbits. Young rabbits grow exceptionally fast, and such tether-related retardation is not surprising. In contrast, in a large animal model using calves, Martel et al. found no evidence of significant growth retardation, although the tethers were only left in for three months [10]. In the only human study, Metaizeau et al. suggested that in their 20 cases there was a mean of 12 mm growth retardation over the course of 2 years of rotationally guided growth [2]. In this study, we did not find any leg length difference between sides in the two bilateral femoral rotationally guided growth cases. The other three patients all had leg length discrepancies to start, so it would be difficult to know if any additional growth inhibition occurred. There were no secondary angular deformities in the frontal or sagittal planes after correction. Two pre-existing angular deformities were corrected by converting the bilaterally guided growth into a unilateral hemiepiphysiodesis (Figure 6). The total time for correction ranged from 7–18 months, which is very similar to the time taken for angular-guided growth in the frontal or sagittal planes. The degree of correction was judged clinically and not radiographically. The correction achieved in the femur ranged from 20° to 35°. Additionally, the correction achieved in the tibia ranged from 5° to 17° (Figure 7). The foot progression angle returned to normal, and all parents and patients were satisfied with the improvement. There were no patients needed to be considered for torsional correction by osteotomy.

## 5. Conclusions

Counter-opposed, inclined peripheral flexible tethers are an effective method to treat rotational malalignment in growing children. Further follow-up and a larger patient cohort will be needed to study the longer-term results and risk for rebound effects. While this study did not show evidence of growth retardation, we cannot rule out that some growth retardation may occur, as was seen in small animals.

## Figures and Tables

**Table 1:** Demographics and rotation profiles before and after rotationally guided growth.

				Before Treatment						After Treatment						Amount of Correction (IR/ER/TFA)		
				Hip Rotation Internal (IR)		Hip Rotation External (ER)		Thig Foot Axis (TFA)		Hip Rotation Internal		Hip Rotation External		Thig Foot Axis				
Patient	Age at Insertion (Years)	Diagnosis	Site	R	L	R	L	R	L	R	L	R	L	R	L	R	L	Time to Correct (Months)
**1**	10.5	Femur anteversion	Bilateral Femur	60	65	20	20			40	40	50	45			−20/30/-	−25/25/-	15
**2**	5.4	Congenital pseudarthrosis of the tibia with external tibial torsion	Right Tibia					30						19		-/-/−11		18
**3**	7.7	Femur anteversion	Right Tibia					−7						10		-/-/17		11
**3**	8.6	Internal tibial torsion	Right Femur	60		40				50		60				−10/20/-		8
**4**	15.7	Congenital Femoral Deficiency with internal tibial torsion	Left Tibia						−15						−10		-/-/5	
**5**	2.6	Femur anteversion	Bilateral Femur	90	90	0	0			45	45	35	35			−45/35/-	−45/35/-	7

## Data Availability

Not applicable.
